# Leptin administration restores the altered adipose and hepatic expression of aquaglyceroporins improving the non-alcoholic fatty liver of *ob/ob* mice

**DOI:** 10.1038/srep12067

**Published:** 2015-07-10

**Authors:** Amaia Rodríguez, Natalia R. Moreno, Inmaculada Balaguer, Leire Méndez-Giménez, Sara Becerril, Victoria Catalán, Javier Gómez-Ambrosi, Piero Portincasa, Giuseppe Calamita, Graça Soveral, María M. Malagón, Gema Frühbeck

**Affiliations:** 1 Metabolic Research Laboratory, Clínica Universidad de Navarra, Pamplona, Spain; 2 Department of Endocrinology & Nutrition, Clínica Universidad de Navarra, Pamplona, Spain; 3 Department of Cell Biology, Physiology, and Immunology, Instituto Maimónides de Investigación Biomédica (IMIBIC)/Reina Sofia University Hospital/University of Córdoba, Córdoba, Spain; 4 Clinica Medica “A. Murri”, Department of Biomedical Sciences and Human Oncology University of Bari Medical School, Policlinico Hospital, Bari, Italy; 5 Department of Biosciences, Biotechnologies and Biopharmaceutics, University of Bari “Aldo Moro”, Bari, Italy; 6 Research Institute for Medicines (iMed.ULisboa), Faculty of Pharmacy, Universidade de Lisboa, Lisboa, Portugal; 7 CIBER Fisiopatología de la Obesidad y Nutrición, Instituto de Salud Carlos III, 28029, Madrid, Spain; 8 Obesity & Adipobiology Group, Instituto de Investigación Sanitaria de Navarra (IdiSNA), Pamplona, Spain

## Abstract

Glycerol is an important metabolite for the control of lipid accumulation in white adipose tissue (WAT) and liver. We aimed to investigate whether exogenous administration of leptin improves features of non-alcoholic fatty liver disease (NAFLD) in leptin-deficient *ob/ob* mice via the regulation of AQP3 and AQP7 (glycerol channels mediating glycerol efflux in adipocytes) and AQP9 (aquaglyceroporin facilitating glycerol influx in hepatocytes). Twelve-week-old male wild type and *ob/ob* mice were divided in three groups as follows: control, leptin-treated (1 mg/kg/d) and pair-fed. Leptin deficiency was associated with obesity and NAFLD exhibiting an AQP3 and AQP7 increase in WAT, without changes in hepatic AQP9. Adipose *Aqp3* and hepatic *Aqp9* transcripts positively correlated with markers of adiposity and hepatic steatosis. Chronic leptin administration (4-weeks) was associated with improved body weight, whole-body adiposity, and hepatosteatosis of *ob/ob* mice and to a down-regulation of AQP3, AQP7 in WAT and an up-regulation of hepatic AQP9. Acute leptin stimulation *in vitro* (4-h) induced the mobilization of aquaglyceroporins towards lipid droplets (AQP3) and the plasma membrane (AQP7) in murine adipocytes. Our results show that leptin restores the coordinated regulation of fat-specific AQP7 and liver-specific AQP9, a step which might prevent lipid overaccumulation in WAT and liver in obesity.

Non-alcoholic fatty liver disease (NAFLD) comprises a spectrum of liver disorders ranging from non-alcoholic fatty liver (NAFL) and non-alcoholic steatohepatitis (NASH) to NASH-cirrhosis, and even hepatocellular carcinoma^1^,^2^. Obesity is a common well-documented risk factor for NAFLD and NASH^3^,^4^,^5^. The prevalence of NAFLD and NASH increases from around 20% and 3%, respectively, in the general population to 75% and 25–70%, respectively, in morbid obesity^3^,^6^. One of the main contributors leading to obesity-associated NAFLD is the increased adipose tissue lipolysis, the catabolic process leading to hydrolysis of triacylglycerols (TG) into free fatty acids (FFA), and glycerol-3-phosphate^7^. FFA released from visceral adipose tissue are collected into the portal vein and reach the liver at high concentrations, a step leading to excessive hepatic TG deposition and, ultimately, hepatocellular damage^8^. However, scarce is the attention on the relevance of hepatic import of glycerol, the other primary source (as glycerol-3-phosphate) of increased TG in hepatocytes^9^.

Aquaglyceroporins (AQP3, 7, 9 and 10) are channel-forming integral membrane proteins that facilitate the movement of water and also small solutes, such as glycerol and urea, across cell membranes^9^,^10^. AQP7 is the main gateway facilitating glycerol release from adipocytes^10^,^11^, although other glycerol channels such as AQP3, 9, 10 and the most recently described AQP11, also contribute to glycerol efflux from fat depots^12^,^13^,^14^. Circulating plasma glycerol is then introduced in hepatocytes by the liver-specific AQP9, where glycerol kinase (GK) catalyzes the initial step for its conversion into glucose (gluconeogenesis) and/or TG^15^,^16^,^17^. Thus, the coordinated regulation of aquaglyceroporins in adipocytes and hepatocytes plays a key role in maintaining the control of fat accumulation in adipose tissue and liver, as well as whole-body glucose homeostasis^9^,^18^,^19^. In this regard both obesity and NAFLD are associated with a dysregulation of aquaglyceroporins in adipose tissue and liver. Obese subjects exhibit high expression of AQP3 and AQP7 in visceral fat and low AQP7 levels in subcutaneous adipose tissue, a condition reflecting increased lipolysis and adipose tissue hypertrophy, respectively, in these fat depots^12^,^19^,^20^,^21^. On the other hand, NAFLD is associated with a down-regulation of AQP9 in experimental animals^22^ and obese patients^23^, suggesting a compensatory mechanism whereby liver prevents further TG accumulation and reduces hepatic gluconeogenesis.

Leptin is an adipocyte-derived hormone that exerts lipolytic effects by counteracting the adenosine deaminase-induced tonic inhibition^24^. Previous *in vitro* studies of our group have shown that leptin repressed AQP7 expression in differentiated human adipocytes via PI3K/Akt/mTOR signalling, suggesting a negative feedback regulation in lipolytic states to limit glycerol release from fat cells^12^. Notably, chronic leptin treatment reverts hepatic steatosis in patients with severe lipodystrophy by stimulating lipolysis in hepatocytes^25^,^26^. Thus, the aim of the present study was to analyze whether the beneficial effects of chronic leptin administration *in vivo* on hepatosteatosis are mediated via the coordinated regulation of aquaglyceroporins in adipose tissue and liver in wild type and leptin-deficient *ob/ob* mice.

## Results

### Acute leptin treatment *in vitro* regulates the expression and intracellular distribution of aquaglyceroporins in murine adipocytes

**Figure 1:**
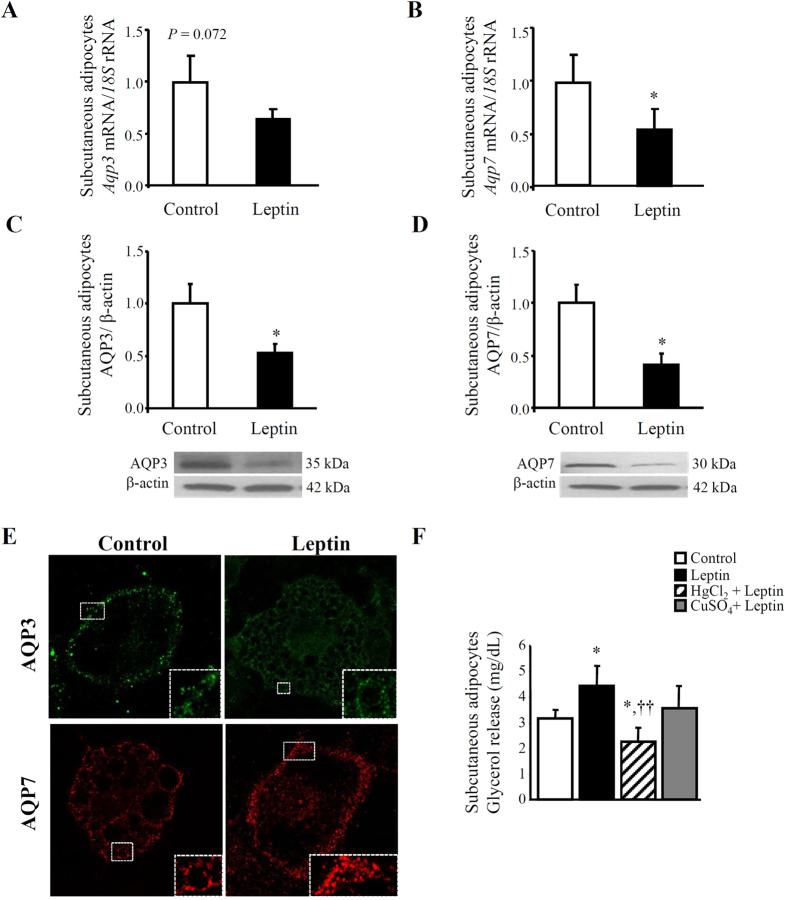
Effect of *in vitro* acute leptin treatment on aquaglyceroporins AQP3 and AQP7 expression and subcellular localization in murine adipocytes. Bar graphs show transcript and protein levels of AQP3 (**A**, **C**) and AQP7 (**B**, **D**) in differentiated murine adipocytes obtained from subcutaneous white adipose tissue (WAT) of wild type mice under basal conditions and after leptin (10 nmol/L) treatment for 24 h. The gene and protein expression in unstimulated cells was assumed to be 1. Representative blots are shown at the bottom of the figure. (**E**) Immunocytochemical detection of the AQP3 and AQP7 proteins in differentiated murine 3T3-L1 adipocytes (day 10) under basal conditions (*upper panels*) and after the stimulation for 4 h with leptin (10 nmol/L) (*lower panels*). Images were taken from the basal planes of the cells. Representative images of at least three separate experiments are shown. (**F**) Glycerol release from murine subcutaneous adipocytes under basal conditions (control) and after leptin (10 nmol/L)-induced stimulation without or with preincubation with HgCl_2_ (0.3 mmol/L), a nonspecific AQP inhibitor, or with CuSO_4_ (0.1 mmol/L), a selective AQP3 inhibitor. Differences between groups were analyzed by Student’s *t* test or one-way ANOVA followed by Tukey’s test. *P < 0.05 *vs*. control unstimulated cells; ††*P* < 0.01 *vs*. adipocytes stimulated with leptin.

Acute leptin treatment increases lipolysis, leading to FFA and glycerol release from the adipose tissue^24^,^27^. We^12^ and others^28^,^29^,^30^ have reported that aquaglyceroporins AQP3 and AQP7 facilitate glycerol outflow from adipocytes in response to the lipolysis induced by the β-adrenergic agonist isoproterenol. Thus, in the present study, the direct effect of acute leptin treatment on aquaglyceroporin expression was analyzed by real-time PCR and Western blot in murine differentiated subcutaneous adipocytes. Upon 24-h leptin stimulation, *Aqp3* mRNA tended to decrease (*P* = 0.072) and *Aqp7* gene expression was down-regulated (*P* < 0.05) in murine subcutaneous adipocytes (Fig. 1A,B). Moreover, both AQP3 and AQP7 protein levels were reduced (*P* < 0.05) after leptin treatment (Fig. 1C,D). To gain more insight into the regulation of aquaglyceroporins by leptin, the subcellular localization of AQP was studied in differentiated 3T3-L1 adipocytes by confocal immunofluorescence microscopy (Fig. 1E). We previously described that after subcellular fractionation of quiescent 3T3-L1 adipocytes, AQP3 was located in the plasma membrane and cytosolic fraction, whereas AQP7 was expressed in the subfractions of lipid droplets and the rest of the cytoplasm^12^. In the present study, we confirmed that, under basal conditions, AQP3 was present mainly in the cell surface, although some punctuate labelling in the cytoplasm could also be observed, while AQP7 resided predominantly in the cytoplasm, surrounding lipid droplets of differentiated 3T3-L1 adipocytes. After 4-h leptin stimulation, AQP3 tended to surround lipid droplets more prominently, whereas AQP7 was translocated to the plasma membrane.

In order to test the functionality of aquaglyceroporins on the lipolytic effect triggered by leptin, murine subcutaneous adipocytes were exposed to leptin 10 nmol/L for 24 h in the presence of HgCl_2_, a nonspecific AQP inhibitor^31^, or to CuSO_4_, a more selective AQP3 inhibitor^32^, prior to determination of glycerol release to the culture media. The inhibition of AQP permeability with 0.3 mmol/L HgCl_2_ alone induced a modest decrease in glycerol release in murine subcutaneous adipocytes (control 3.18 ± 0.19 *vs*. HgCl2 3.06 ± 0.30 mg/dL, *P* = 0.729). Nonetheless, mercury ions abolished around 50% of the leptin-induced glycerol release in murine subcutaneous adipocytes, while copper ions inhibited approximately 20% of the glycerol release caused by leptin (Fig. 1F). These data suggest that the major glycerol channel in murine adipocytes, AQP7 and, to a lesser extent, AQP3 mediate the glycerol efflux triggered by leptin in fat cells.

### Chronic leptin administration *in vivo* reduces adiposity in parallel to a decrease in aquaglyceroporins AQP3 and AQP7 in adipose tissue

Leptin is an adipokine that reduces food intake and increases energy expenditure to maintain energy balance^33^. As expected, leptin-deficient *ob/ob* mice exhibited severe obesity and hyperphagia (Table 1). Chronic leptin treatment corrected the obese phenotype of *ob/ob* mice, as evidenced by the lower body weight as well as epididymal, subcutaneous and perirenal fat mass via the reduction of food intake and the increase in rectal temperature. In the present study, chronic leptin administration was associated with a decrease in circulating FFA and glycerol, pointing to a lower lipolytic rate in leptin-treated animals.

**Figure 2:**
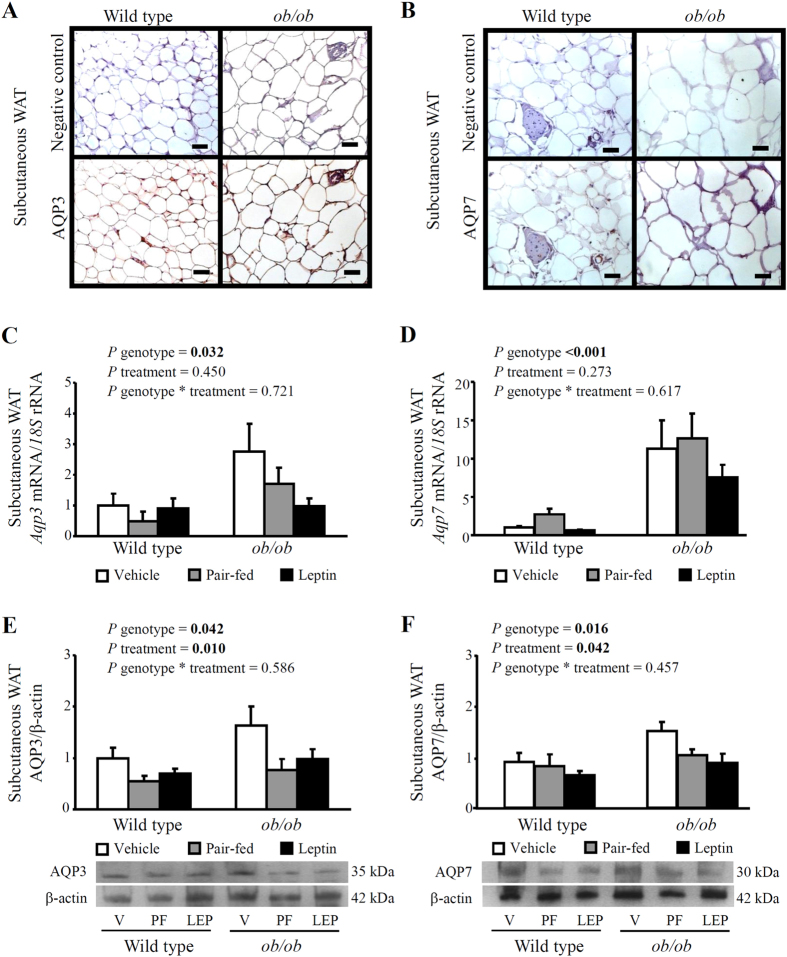
Effect of *in vivo* chronic leptin administration on aquaglyceroporins AQP3 and AQP7 expression and tissue distribution in adipose tissue of wild type and *ob/ob* mice. Immunohistochemical detection of AQP3 (**A**) and AQP7 (**B**) in subcutaneous white adipose tissue (WAT) of wild type (*left panels*) and *ob/ob* (*right panels*) mice (magnification 200X, scale bar = 50 μm). Bar graphs show transcript and protein levels of AQP3 (**C**, **E**) and AQP7 (**D**, **F**) in subcutaneous WAT obtained from vehicle-treated, pair-fed and leptin-treated wild type and *ob/ob* mice. The gene and protein expression in vehicle-treated wild type mice was assumed to be 1. Representative blots are shown at the bottom of the figure. Differences between groups were analyzed by two-way ANOVA.

To analyze the potential involvement of aquaglyceroporins in the changes observed on adiposity after chronic exogenous leptin administration (4 weeks), we first assessed the gene and protein expression of AQP3 and AQP7 in subcutaneous WAT of the experimental groups by real-time PCR, Western blot and immunohistochemistry (Fig. 2). As illustrated in Fig. 2A,B, the tissue distribution of AQP3 and AQP7 showed a predominant immunostaining in the stromovascular fraction and lower expression in mature adipocytes, as previously reported by our group and others^12^,^34^. In the multiple lineal regression analysis, AQP3 and AQP7 protein levels in subcutaneous WAT contributed independently to 51.0% (*P* < 0.05) and 51.2% (*P* < 0.05) to the circulating glycerol concentrations after controlling for body weight, suggesting an important role of these aquaglyceroporins in glycerol efflux from adipose tissue.

Leptin deficiency was associated with higher mRNA and protein levels of AQP3 and AQP7 in subcutaneous WAT (Fig. 2C–F). In line with these results, *Aqp3* and *Aqp7* mRNA levels were positively associated with markers of adiposity [body weight (r = 0.33, *P* = 0.025 and r = 0.44, *P* = 0.001) or subcutaneous WAT/body weight (r = 0.33, *P* = 0.025 and r = 0.53, *P* = 0.001)] and hepatosteatosis [liver/body weight (r = 0.36, *P* = 0.013 and r = 0.35, *P* = 0.010 and intrahepatic TG (r = 0.40, *P* = 0.006 and r = 0.53, *P* < 0.001)]. No differences in the transcript levels of *Aqp3* and *Aqp7* were detected after leptin administration, but a tendency towards a down-regulation of both glycerol channels was observed in leptin-treated *ob/ob* mice. Nonetheless, at the protein level, both leptin administration and caloric restriction reduced (*P* < 0.05) AQP3 and AQP7 in subcutaneous WAT of wild type and *ob/ob* mice.

### Exogenous leptin replacement reduces the hepatic steatosis of *ob/ob* mice and upregulates AQP9 expression in the liver

**Figure 3:**
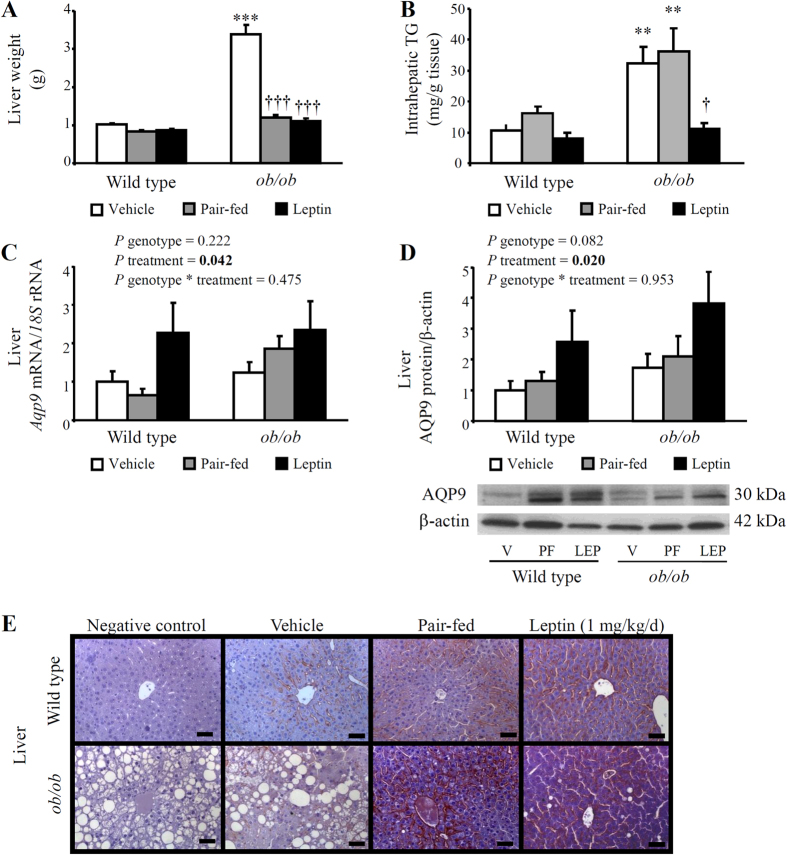
Effect of *in vivo* chronic leptin administration on fatty liver and hepatic aquaglyceroporin AQP9 expression in experimental animals. Bar graphs show the liver weight (**A**) intrahepatic concentrations of triacylglycerols (TG) (**B**) as well as gene (**C**) and protein (**D**) expression levels of AQP9 in the liver of vehicle-treated, pair-fed and leptin-treated wild type and *ob/ob* mice. The gene and protein expression in vehicle-treated wild type mice was assumed to be 1. Representative blots are shown at the bottom of the figure. (**E**) Inmunohistochemical detection of AQP9 protein in histological sections of liver of wild type (*upper panels*) and *ob/ob* mice (*lower panels*) (magnification, 200X; scale bar, 50 μm). Differences between groups were analyzed by two-way ANOVA or one-way ANOVA followed by Tukey’s *post-hoc* test, if an interaction was detected. **P < 0.01; ***P < 0.001 *vs*. vehicle-treated wild type mice; †*P* < 0.05; †††*P* < 0.001 *vs*. vehicle-treated *ob/ob* mice.

Leptin-deficient *ob/ob* mice showed an increased (*P* < 0.0001) liver weight that was significantly reduced (*P* < 0.0001) by either caloric restriction or leptin replacement (Fig. 3A). Histological sections of leptin-deficient *ob/ob* mice were characterized by the presence of severe macrovesicular steatosis, but not advanced inflammation/fibrosis, that was completely reverted after leptin administration for 28 days (Fig. 3E). The analysis of intrahepatic triacylglycerol content revealed elevated TG levels (*P* < 0.001) in the liver of *ob/ob* mice that was prevented by leptin treatment (*P* < 0.05), but not by caloric restriction (Fig. 3B).

We next analyzed the expression of AQP9, the primary route for glycerol uptake in murine hepatocytes, by real-time PCR, Western blot and immunohistochemistry. As previously described by our group^22^, two immunoreactive bands of 30–32 kDa, corresponding to the core and N-glycosylated forms of AQP9 protein, respectively, were observed in the immunoblots (Fig. 3D). Leptin deficiency was associated with similar expression of *AQP9* mRNA and whole (glycosylated and non-glycosylated) AQP9 protein signal than that observed in wild type mice, with leptin administration and caloric restriction increasing (*P* < 0.05) AQP9 gene and protein expression (Fig. 3C,D). *Aqp9* gene expression was positively associated with markers of adiposity [body weight (r = 0.60, *P* < 0.001) or subcutaneous WAT/body weight (r = 0.44, *P* = 0.002)] and hepatic steatosis [liver/body weight (r = 0.69, *P* < 0.001) and intrahepatic TG (r = 0.28, *P* < 0.05)]. Liver sections showed a strong immunoreactivity for AQP9 after leptin infusion, which was mainly localized in the plasma membrane of hepatocytes around the central veins (Fig. 3E).

### Positive association of PPARγ with changes observed in the expression of aquaglyceroporins in adipose tissue and liver after leptin replacement

**Figure 4:**
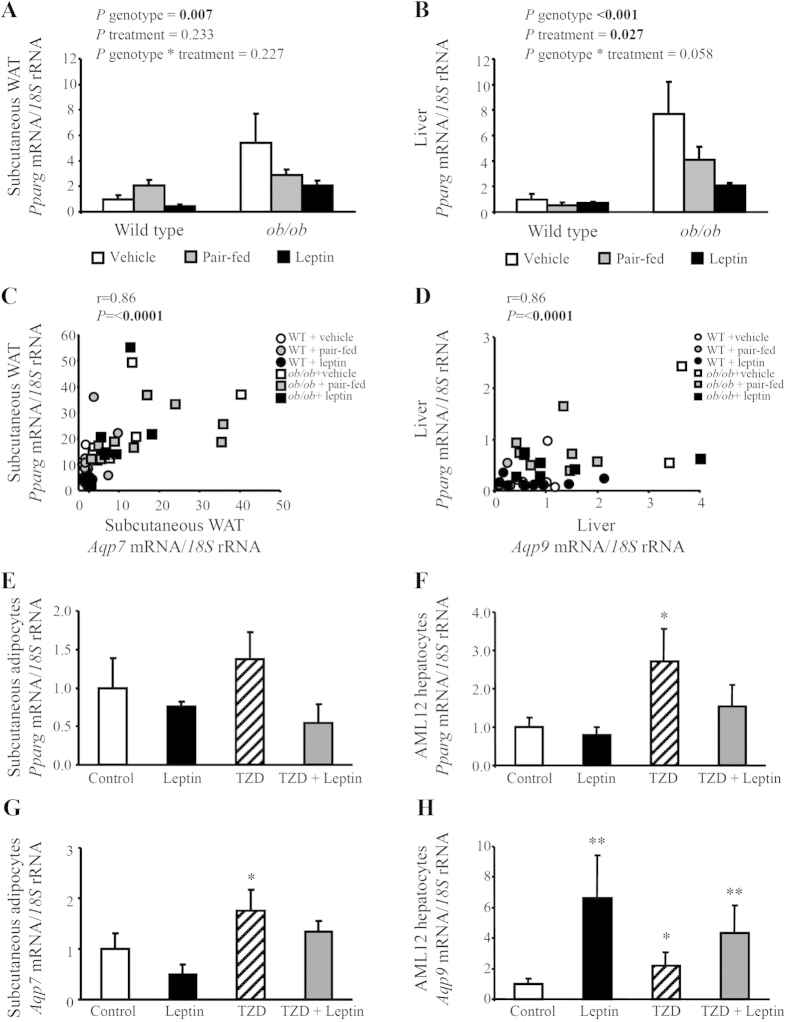
Positive association of *Pparg* transcript levels with aquaglyceroporins *Aqp7* and *Aqp9* in adipose tissue and liver of experimental animals. Bar graphs illustrate the changes in *Pparg* mRNA levels in subcutaneous white adipose tissue (WAT) (**A**) and liver (**B**) obtained from vehicle-treated, pair-fed and leptin-treated wild type and *ob/ob* mice. A positive correlation was found between *Pparg* and *Aqp7* mRNA in subcutaneous WAT (**C**) as well as between *Pparg* and *Aqp9* transcript levels in liver (**D**) of experimental groups. The Pearson’s correlation coefficient (r) and *P* values are indicated. Effect of leptin stimulation for 24 h on basal and thiazolidinedione (TZD) rosiglitazone (10 μmol/L)-induced expression of *Pparg* (**E**, **F**) and aquaglyceroporins *Aqp7* and *Aqp9* (G, H) in murine differentiated subcutaneous adipocytes and AML12 hepatocytes. The gene expression in vehicle-treated wild type mice or unstimulated cells was assumed to be 1. Differences between groups were analyzed by two-way ANOVA or one-way ANOVA followed by Tukey’s *post-hoc* test, where appropriate. *P < 0.05; **P < 0.01 vs. control unstimulated cells.

Peroxisome proliferator-activated receptor γ (PPARγ) represents a well-known lipogenic factor and, importantly, putative peroxisome proliferator response elements (PPRE) are present in the promoters of *Aqp3* and *Aqp7* genes^35^,^36^. In line with the observed excess adiposity and hepatic steatosis, leptin-deficient mice exhibited higher *Pparg* mRNA levels in the adipose tissue and liver that were reduced by leptin replacement and, to a lesser extent, by caloric restriction (Fig. 4A,B). As expected, gene expression levels of *Pparg* in subcutaneous WAT and liver were positively associated with markers of obesity [body weight (r = 0.43, *P* < 0.001 and r = 0.70, *P* < 0.0001) or subcutaneous WAT/body weight (r = 0.35, *P* = 0.010 and r = 0.70, *P* < 0.0001)], fatty liver [liver weight/body weight (r = 0.43, *P* < 0.001 and r = 0.65, *P* < 0.0001) and intrahepatic TG (r = 0.40, *P* = 0.003 and r = 0.45, *P* = 0.001)]. Moreover, a strong positive association was found between *Pparg* transcript levels and *Aqp7* mRNA in the adipose tissue as well as with *Aqp9* mRNA in the liver (Fig. 4C,D). *Pparg* mRNA was also correlated with *Aqp3* gene expression in subcutaneous WAT but to a lower extent (r = 0.43, *P* < 0.001).

To gain further insight into the plausible association of PPARγ with these glycerol channels after leptin treatment, we examined the effect of leptin stimulation on basal and PPARγ agonist rosiglitazone-induced expression of aquaglyceroporins in murine subcutaneous differentiated adipocytes and AML12 hepatocytes. As expected, rosiglitazone stimulation for 24 h upregulated 1.4- and 2.0-fold the transcription of *Pparg* gene in murine subcutaneous adipocytes and AML12 hepatocytes, respectively, although no statistical differences between groups were found in fat cells (*P* = 0.269) (Fig. 4E,F). Moreover, the treatment with this TZD also increased the transcription of *Aqp7* in subcutaneous fat cells and of *Aqp9* in AML12 hepatocytes (Fig. 4G,H). The co-incubation with leptin tended to reduce both basal and TZD-induced mRNA expression of *Pparg* and *Aqp7* genes in subcutaneous adipocytes, although changes fell out of statistical significance (*P* = 0.083 and *P* = 0.125, respectively). A similar trend was observed for the effect of leptin on basal and rosiglitazone-induced expression of *Aqp3* gene in subcutaneous adipocytes (control 1.0 ± 0.4 A.U.; leptin 0.4 ± 0.1 A.U.; TZD 4.3 ± 1.6 A.U.; TZD + leptin 2.7 ± 0.8 A.U.; *P* = 0.003). However, leptin co-treatment induced a slight down-regulation of *Pparg* transcript levels (*P* = 0.292), while increasing (*P* < 0.05) the transcription of *Aqp9* in AML12 hepatic cells.

## Discussion

Adipocyte lipolysis is the process that controls the breakdown of TG into glycerol and FFA, which are released into the circulation and used as energy substrates in metabolic organs^7^,^37^. AQP3 and AQP7 facilitate glycerol outflow from adipocytes in response to β-adrenergic receptor-stimulated lipolysis via its translocation from the cytosolic fraction (AQP3) or lipid droplets (AQP7) to the plasma membrane^12^,^28^,^29^. Basal lipolytic activity of adipocytes is conditioned not only by catecholamines, but also by other factors, such as atrial natriuretic peptides, insulin, leptin, adenosine, tumor necrosis factor α (TNF-α) or neuropeptide Y, among others^7^. The adipokine leptin exerts an autocrine/paracrine lipolytic effect on murine adipocytes^27^. In this sense, acute leptin treatment (1 h) reportedly increases basal lipolysis of wild type and *ob/ob* mice^27^. Here, we found that acute leptin treatment (4 h) stimulated AQP3 translocation from the plasma membrane to lipid droplets, a step that might reflect the glycerol efflux from lipid droplets after lipolytic response in differentiated subcutaneous murine adipocytes. Upon leptin stimulation, AQP7 was translocated from lipid droplets to the plasma membrane, and this finding suggests that this glycerol channel constitutes the main gateway for glycerol secretion to the bloodstream. Thus, we speculate that acute leptin treatment induces the translocation of AQP3 and AQP7 to lipid droplets and the plasma membrane, respectively, to facilitate glycerol mobilization after lipolysis. Nonetheless, the existence of further operative glycerol channels in subcutaneous adipocytes cannot be discarded.

**Figure 5:**
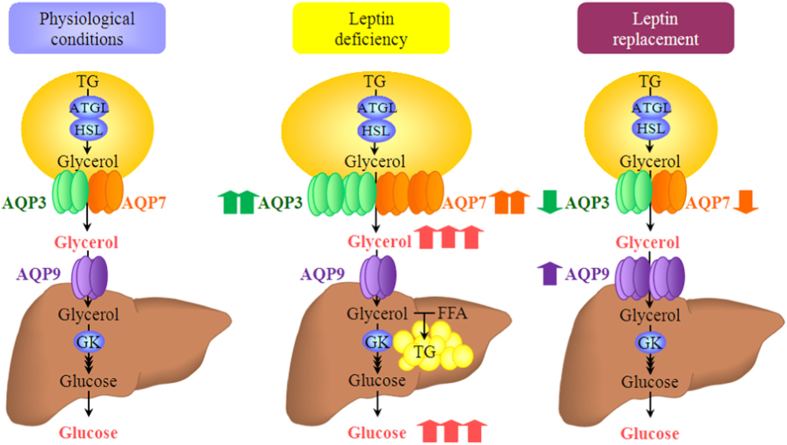
Proposed working model for the coordinated regulation of aquaglyceroporins in adipose tissue and liver by leptin.

Obesity is associated with increased lipolysis due to higher lipolytic activity of β_3_-adrenergic receptors and reduced anti-lipolytic action of insulin, leading to elevated circulating concentrations of FFA and glycerol^38^,^39^. In the present study, we found that leptin-deficient obese *ob/ob* mice showed increased circulating glycerol together with higher subcutaneous fat expression of AQP3 and AQP7. Both chronic leptin treatment and caloric restriction significantly decreased circulating glycerol and AQP3 and AQP7 proteins in subcutaneous adipose tissue in *ob/ob* mice. The adipose tissue is composed not only by adipocytes, but also by SVFCs (i.e., macrophages, T lymphocytes, endothelial cells, fibroblasts, vascular smooth muscle cells or mesenchymal stem cells). Because SVFCs might contribute to the reduction of aquaglyceroporins in adipose tissue, we also studied the direct effect of leptin treatment on differentiated murine subcutaneous adipocytes. In line with the results obtained with the whole adipose tissue, 24-h leptin treatment decreased the gene and protein expression of AQP3 and AQP7 of differentiated murine subcutaneous adipocytes. In this regard, in a previous study, we found that *in vitro* 24-h leptin treatment downregulated AQP7 protein expression in differentiated human adipocytes via the PI3K/Akt/mTOR signalling pathway^12^. Taken together, both *in vivo* chronic leptin administration and caloric restriction limit glycerol release from adipocytes through the down-regulation of AQP3 and AQP7, suggesting a negative feedback regulation in lipolytic states to maintain intracellular glycerol and, therefore, to avoid the depletion of fat stores (Fig. 5).

Liver steatosis is a multi-factorial disease where abnormal TG accumulation in the hepatocytes can be triggered by metabolic, toxic, pharmacological or viral insults across a genetic predisposition^1^,^2^. Glycerol-3-phosphate constitutes a key metabolite for *de novo* synthesis of TG and derives from glycolysis, glyceroneogenesis as well as recycling of glycerol by GK^40^,^41^. AQP9 represents the main facilitative pathway for glycerol uptake as a substrate for gluconeogenesis and lipogenesis in hepatocytes^15^,^16^,^17^. Interestingly, a decrease in hepatic AQP9 and glycerol permeability has been observed in murine and human NAFLD, suggesting a defensive mechanism to prevent further development of hyperglycemia and hepatosteatosis^19^,^22^,^23^. Moreover, a dysregulation of AQP9 has been observed in several hepatic inflammatory derangements, such as extrahepatic cholestasis, alcoholic steatohepatitis and NASH^23^,^42^,^43^,^44^. However, little is known about the regulation of AQP9 in the context of NAFLD/NASH. In the present study, AQP9 was mainly localized in the sinusoidal domain of the plasma membrane of hepatocytes, which is in agreement with previous results^45^,^46^ including ours^12^,^23^. Leptin-deficient mice, a murine model of NAFLD, displayed macrovesicular steatosis without changes in hepatic AQP9 mRNA and protein. In a previous study, a lower expression of AQP9 was found in liver samples of *ob/ob* mice^22^. In this regard, AQP9 expression in the liver is influenced by the degree of hepatic steatosis and inflammation^23^ that might change the expression of this aquaglyceroporin during the ongoing NAFLD in adult *ob/ob* mice. Short-term leptin administration has been reported to exert profound effects on hepatic lipid metabolism of *ob/ob* mice by reducing *de novo* lipogenesis via repressing acetyl-CoA carboxylase (ACC), fatty acid synthase (FAS) or stearoyl-coenzyme A desaturase 1 (SCD1) expression, and through the activation of β-oxidation by increasing the transcript levels of acetyl-coenzyme A acetyl-transferase 1 (ACAT1) or carnitine palmitoyl transferase 1 (CPT1)^47^. We herein show that chronic leptin administration completely rescues the hepatosteatosis of *ob/ob* mice as evidenced by the normalization of intrahepatocellular hepatocytes and liver morphology. Moreover, a valuable result of this work regards the up-regulation of AQP9 after chronic leptin treatment in wild type and *ob/ob* mice. Taken together, similar or lower levels of AQP9 associated to leptin deficiency appear to reflect a defensive cell reaction of the steatotic hepatocyte. Interestingly, chronic leptin administration not only rescues the fatty liver, but also increases AQP9 in order to facilitate glycerol import into hepatocytes for maintaining the glycemia as well as an appropriate lipid metabolism.

The adipose tissue and liver from leptin-deficient *ob/ob* mice showed an induction of PPARγ, which is a critical transcription factor for the development of obesity and hepatic steatosis as previously reported by other authors^48^,^49^,^50^. In a previous study of our group^51^, we found that the downregulation of PPARγ in adipose tissue and liver of diet-induced obese rats after bariatric surgery was strongly associated with a reduction in the transcription of aquaglyceroporins in these tissues. Nonetheless, the molecular mechanisms underlying this association were unclear. In the present study, we found that chronic leptin administration significantly decreased *Pparg* transcript levels in parallel with the improvement of adiposity and fatty liver. Interestingly, the promoters of *Aqp3* and *Aqp7* genes present putative PPRE with the expression of these aquaglyceroporins being up-regulated by PPARγ agonists^35^,^36^. In line with this observation, *Pparg* transcript levels were positively correlated with *Aqp3* and *Aqp7* in adipose tissue, but also with *Aqp9* in the liver. Moreover, leptin co-treatment tended to reduce the transcription of PPARγ and AQP7 induced by rosiglitazone stimulation, a well-known PPARγ-selective agonist, in murine subcutaneous adipocytes. Our results are in agreement with other reports showing that pioglitazone and rosiglitazone administration to rodents increase the expression of AQP7 in adipose tissue^35^,^52^. However, leptin increased both basal and rosiglitazone-induced transcription of *Aqp9* in AML12 hepatocytes, despite inducing a slight reduction *Pparg* mRNA levels in these hepatic cells. Thus, the mild action of leptin on rosiglitazone-induced up-regulation of aquaglyceroporins in adipocytes and hepatocytes suggests that other upstream molecules in addition to PPARγ might be involved in the regulatory effect of this adipokine.

The coordinated regulation of adipose and hepatic aquaglyceroporins is extremely relevant to maintain the control of fat accumulation and glycemia (Fig. 5)^12^,^18^. We herein report, for the first time, that chronic leptin administration regulates the altered expression of the adipose glycerol channels AQP3 and AQP7 and the liver-specific AQP9 in leptin-deficient obese *ob/ob* mice. Since glycerol is a key metabolite for lipid accumulation in fat depots and liver, the improvement of glycerol availability might be involved in the beneficial effects of leptin on obesity and NAFLD. Nonetheless, future *in vivo* studies are needed to fully demonstrate the requirement of AQP proteins for the improvement of these pathologies. Moreover, the time functional link between the regulation of AQP and leptin-dependent changes in lipid flux at the clinical level require the exact characterization of NAFLD and more advanced liver damage stages in patients with respect to weight changes and diet.

## Methods

### Animals

Ten-week-old male wild type (C57BL/6J) (n = 30) and genetically obese *ob/ob* mice (C57BL/6J) (n = 30) (Harlan Laboratories Inc., Barcelona, Spain) were housed in a room with controlled temperature (22 ± 2 °C), and a 12:12 light-dark cycle (lights on at 08:00 am). Wild type and *ob/ob* mice were divided in control, leptin-treated (1 mg/kg/d) and pair-fed groups (n = 10 per group), as previously described^26^. The control and pair-fed groups received vehicle (PBS), while leptin-treated groups were intraperitoneally administered with leptin (Bachem, Bubendorf, Switzerland) twice a day at 08:00 and 20:00 for 28 days. Control and leptin-treated groups were provided with water and food *ad libitum* with a rodent maintenance diet (12.1 kJ: 4% fat, 82% carbohydrate and 14% protein, Diet 2014S, Teklad Global Diets, Harlan, Barcelona, Spain), while the daily food intake of the pair-fed groups was matched to the amount eaten by the leptin-treated groups the day before to discriminate the inhibitory effect of leptin on appetite. All experimental groups had an isoproteic intake consuming similar amounts of sodium and phytates^53^. Body weight and food intake were daily registered and rectal temperature was measured using a thermoprobe (YSI 4600 Series Precision Thermometers, YSI Temperature, Dayton, OH, USA) at the end of the experiment. Animals were sacrificed on the 28^th^ day of treatment by CO_2_ inhalation. Epididymal, subcutaneous and perirenal white adipose tissue (WAT) as well as the liver were rapidly dissected out, weighed, frozen in liquid nitrogen, and stored at −80 °C until mRNA and protein extraction. A piece of the tissues was fixed in 4% formaldehyde for immunohistochemical analyses. All experimental procedures conformed to the European Guidelines for the Care and Use of Laboratory Animals (directive 2010/63/EU) and were approved by the Ethical Committee for Animal Experimentation of the University of Navarra (041/08).

### Blood and tissue assays

Blood assays were determined as previously described^26^. Intrahepatic TG content was measured by enzymatic methods, in accordance with previously published procedures^12^. Briefly, liver biopsies were homogenized and diluted in saline at a final concentration of 50 mg/mL. Homogenates were diluted (1:1) in 1% deoxycholate (Sigma, St. Louis, MO, USA) and incubated at 37 °C for 5 min. For TG measurements, samples were diluted 1:100 in the reagent (Infinity^™^ Triglycerides Liquid Stable Reagent, Thermo Electron Corporation, Melbourne, Australia) and incubated for 30 min at 37 °C. The resulting dye was measured based on its absorbance at 550 nm. Concentrations were determined compared with a standard TG curve (Infinity^™^ Triglycerides Standard, Thermo Electron Corporation). The protein content of the preparations was measured by the Bradford method, using bovine serum albumin (BSA) (Sigma) as standard. All assays were performed in duplicate.

### RNA extraction and real-time PCR

RNA isolation and purification was performed as described earlier^19^. Transcript levels for *Aqp3* (NM_016689.2), *Aqp7* (NM_007473.4), *Aqp9* (NM_022026.2) and *Pparg* (NM_001127330.1) were quantified by real-time PCR (7300 Real Time PCR System, Applied Biosystems, Foster City, CA, USA). Primers and probes (Supplementary Table S1) were designed using the software Primer Express 2.0 (Applied Biosystems) and purchased from Genosys (Sigma). Primers or TaqMan^®^ probes encompassing fragments of the areas from the extremes of two exons were designed to ensure the detection of the corresponding transcript avoiding genomic DNA amplification. The cDNA was amplified at the following conditions: 95 °C for 10 min, followed by 45 cycles of 15 s at 95 °C and 1 min at 59 °C, using the TaqMan^®^ Universal PCR Master Mix (Applied Biosystems). The primer and probe concentrations were 300 and 200 nmol/L, respectively. All results were normalized for the expression of 18 S rRNA (Applied Biosystems), and relative quantification was calculated using the ΔΔCt formula^19^. Relative mRNA expression was expressed as fold expression over the calibrator sample. All samples were run in triplicate and the average values were calculated.

### Western blot studies

Tissues and cells were harvested and homogenized in ice-cold lysis buffer (0.1% SDS, 1% Triton X-100, 5 mmol/L EDTA∙2 H_2_O, 1 mol/L Tris-HCl, 150 mmol/L NaCl, 1% sodium deoxycholate, pH 7.40) supplemented with a protease inhibitor cocktail (Complete^TM^ Mini-EDTA free, Roche, Mannheim, Germany). Lysates were centrifuged at 16,000 g at 4 °C for 15 min to remove nuclei and unbroken cells. Total protein concentrations were determined by the Bradford assay. Thirty micrograms of total protein were diluted in loading buffer 4X (20% β-mercaptoethanol, 40 mmol/L dithiothreitol, 8% SDS, 40% glycerol, 0.016% bromophenol blue, 200 mmol/L Tris-HCl, pH 6.80) and heated for 10 min at 100 °C. Samples were run out in 10% SDS-PAGE, subsequently transferred to nitrocellulose membranes (Bio-Rad Laboratories, Inc., Hercules, CA, USA) and blocked in Tris-buffered saline (TBS) (10 mmol/L Tris-HCl, 150 mmol/L NaCl, pH 8.00) with 0,05% Tween 20 containing 5% non-fat dry milk for 1 h at room temperature (RT). Blots were then incubated overnight at 4 °C with goat polyclonal anti-AQP3, rabbit polyclonal anti-AQP7, goat polyclonal anti-AQP9 (Santa Cruz Biotechnology, Inc., Santa Cruz, CA, USA) or murine monoclonal anti-β-actin (Sigma) (diluted 1:5,000 in blocking solution). The antigen-antibody complexes were visualized using horseradish peroxidase (HRP)-conjugated anti-goat, anti-rabbit or anti-mouse IgG antibodies (diluted 1:5,000 in blocking solution) and the enhanced chemiluminescence ECL Plus detection system (Amersham Biosciences, Buckinghamshire, UK). The intensity of the bands was determined by densitometric analysis with the Gel Doc^TM^ gel documentation system and Quantity One 4.5.0 software (Bio-Rad) and normalized with β-actin density values. All assays were performed in duplicate.

### Immunohistochemistry

The immunodetection of AQP3, AQP7 and AQP9 in histological sections of subcutaneous adipose tissue and liver was performed by the indirect immunoperoxidase method^12^. Sections of formalin-fixed paraffin-embedded adipose tissue (6 μm) or liver (4 μm) were dewaxed in xylene, rehydrated in decreasing concentrations of ethanol and treated with 3% H_2_O_2_ (Sigma) in absolute methanol for 10 min at RT to quench endogenous peroxidase activity. Slides were blocked during 1 h with 1% goat serum (Sigma) diluted in TBS (50 mmol/L Tris, 0.5 mol/L NaCl; pH 7.36) to prevent nonspecific adsorption. Sections were incubated overnight at 4 °C with goat polyclonal anti-AQP3, rabbit anti-AQP7 (Santa Cruz Biotechnology) or rabbit anti-AQP9 (#AQP91-A, Alpha Diagnostic International, San Antonio, TX, USA) antibodies diluted 1:100 for AQP3 and AQP7 in subcutaneous WAT and 1:500 for AQP9 in liver in TBS. After washing three times (5 min each) with TBS, slides were incubated with HRP-conjugated anti-goat IgG diluted in TBS (1:500) or Dako Real^TM^ EnVision^TM^ HRP-conjugated anti-rabbit/mouse (Dako, Glostrup, Denmark) for 1 h at RT. The peroxidase reaction was visualized using a 0.5 mg/mL diaminobenzidine (DAB)/0.03% H_2_O_2_ solution diluted in 50 mmol/L Tris-HCl, pH 7.36, and Harris hematoxylin solution (Sigma) as counterstaining. Negative control slides without primary antibody were included to assess non-specific staining.

### Cell cultures

Murine stromovascular fraction cells (SVFC) were isolated from subcutaneous adipose tissue from wild type mice as previously described^12^. SVFC were seeded at 2 × 10^5^ cells/cm^2^ and grown in adipocyte medium [DMEM/F-12 [1:1] (Invitrogen, Paisley, UK), 16 μmol/L biotin, 18 μmol/L panthotenate, 100 μmol/L ascorbate and antibiotic-antimycotic] supplemented with 10% newborn calf serum (NCS). After 4 days, the medium was changed to adipocyte medium supplemented with 3% NCS, 0.5 mmol/L 3-isobutyl-1-methylxanthine (IBMX), 0.1 μmol/L dexamethasone, and 10 μg/mL insulin. After a 3-day induction period, cells were fed every 2 days with the same medium but without IBMX supplementation for the remaining 7 days of adipocyte differentiation.

The non-tumorigenic mouse hepatocyte cell line AML12 was purchased from ATCC (Manassas, VA). Murine AML12 hepatocytes were maintained in DMEM/F-12 medium (Invitrogen) supplemented with 10% fetal bovine serum (FBS), 5 μg/mL insulin, 5 μg/mL transferrin, 5 ng/mL selenium (Invitrogen), 40 ng/mL dexamethasone (Sigma), and antibiotic-antimycotic (Complete Growth Medium). AML12 cells were seeded at 2 × 10^5^ cell/cm^2^ and grown in Complete Growth Medium.

Differentiated subcutaneous adipocytes and AML12 hepatocytes were serum-starved for 24 h and quiescent cells were stimulated with recombinant murine leptin (10 nmol/L) (450-31, PeproTech EC, Inc., Rocky Hill, NJ, USA) or with TZD rosiglitazone (BRL49653, Cayman Chemical Ann Arbor, MI) 10 μmol/L for 24 h. One sample per experiment was used to obtain control responses in the presence of the solvent.

### Measurement of glycerol release

Glycerol release to the culture media was evaluated according to previously described methods^12^. Briefly, differentiated murine subcutaneous adipocytes were stimulated with leptin 10 nmol/L for 24 h at 37 °C in the presence or absence of HgCl_2_ (0.3 mmol/L) or CuSO_4_ (0.1 mmol/L). The glycerol concentration in the culture media was measured by a quantitative enzymatic determination assay (Sigma). Intra- and inter-assay coefficients of variation were 1.5% and 4.2%, respectively.

### Confocal immunofluorescence microscopy

3T3-L1 cells were differentiated into adipocytes as previously described^12^, grown on glass coverslips and stimulated with leptin (10 nmol/L) for 4 h. Cells were fixed in cold methanol (−20 °C), washed with PBS, permeabilized with blocking solution (PBS containing 0.1% Triton X-100 and 5 mmol/L glycine) for 1 h at RT and then incubated with goat polyclonal anti-AQP3 or rabbit polyclonal anti-AQP7 (Santa Cruz Biotechnology) antibodies diluted 1:100 overnight at 4 °C. After washing with PBS, cells were incubated with Alexa Fluor^®^ 488-conjugated donkey anti-goat IgG or Alexa Fluor^®^ 594 chicken anti-rabbit (Invitrogen) diluted 1:200 for 2 h at RT. After washing, coverslips were mounted on microscope slides and examined under a TCS-SP2-AOBS confocal laser scanning microscope (Leica Corp., Heidelberg, Germany).

### Statistical analysis

Data are expressed as the mean ± SEM. Statistical differences between mean values were analyzed using two-way ANOVA (genotype x treatment) or one-way ANOVA followed by Tukey’s post-hoc test, if an interaction was detected. Pearson correlation coefficients (r) and stepwise multiple linear regression analysis were used to analyze the association between variables. A *P* value < 0.05 was considered statistically significant. The statistical analyses were performed using the SPSS/Windows version 15.0 software (SPSS Inc,. Chicago, IL, USA).

## Additional Information

**How to cite this article**: Rodríguez, A. *et al*. Leptin administration restores the altered adipose and hepatic expression of aquaglyceroporins improving the non-alcoholic fatty liver of *ob/ob* mice. *Sci. Rep*. **5**, 12067; doi: 10.1038/srep12067 (2015).

## Supplementary Material

Supplementary Information

## Figures and Tables

**Table 1:** Markers of adiposity, body temperature and lipolysis of experimental groups.

	Wild type			*ob/ob*		
	Control	Pair-fed	Leptin	Control	Pair-fed	Leptin
n	9	10	9	10	10	9
Body weight (g)^a,b,c^	25.4 ± 0.2	22.6 ± 0.1^*^	22.6 ± 0.1^*^	49.2 ± 0.8^*^	33.6 ± 0.4^*,†^	24.9 ± 0.2^†,$^
Cumulative food intake (g)^a,b,c^	94 ± 2	88 ± 1	88 ± 1	191 ± 5^*^	66 ± 5^*,†^	66 ± 5^*,†^
Rectal temperature (°C)^c^	35.4 ± 0.2	36.0 ± 0.2	35.4 ± 0.1	35.7 ± 0.2	34.7 ± 0.3^†^	36.0 ± 0.2^$^
Epididymal WAT (g)^a,b,c^	0.19 ± 0.01	0.13 ± 0.02	0.04 ± 0.01	1.64 ± 0.10^*^	1.04 ± 0.05^*,†^	0.46 ± 0.06^*,†,$^
Subcutaneous WAT (g)^a,b,c^	0.17 ± 0.01	0.15 ± 0.02	0.09 ± 0.05	3.07 ± 0.32^*^	1.85 ± 0.11^*,†^	0.47 ± 0.06^*,†,$^
Perirrenal WAT (g)^a,b,c^	0.05 ± 0.01	0.03 ± 0.01	0.01 ± 0.01	0.92 ± 0.05^*^	0.51 ± 0.04^*,†^	0.09 ± 0.01^*,†,$^
Total white adiposity (g)^a,b,c^	0.42 ± 0.04	0.31 ± 0.01	0.14 ± 0.05	5.62 ± 0.38^*^	3.40 ± 0.23^*,†^	1.03 ± 0.13^*,†,$^
FFA (mg/dL)^b^	44 ± 8	45 ± 5	32 ± 4	39 ± 5	55 ± 11	24 ± 2
Glycerol (mmol/mL)^a,b,c^	0.05 ± 0.01	0.03 ± 0.01	0.02 ± 0.01^*^	0.07 ± 0.01^*^	0.05 ± 0.01	0.01 ± 0.01^*,†,$^

FFA, free fatty acids; WAT, white adipose tissue. Values presented as the mean ± SEM. Differences between groups were analyzed by two-way ANOVA or one-way ANOVA followed by Tukey’s *post-hoc* test when an interaction between factors was detected. ^a^*P* < 0.05, effect of genotype; ^b^*P* < 0.05 effect of treatment; ^c^*P* < 0.05 interaction between genotype and treatment. ^*^P < 0.05 *vs*. vehicle-treated wild type mice; ^†^*P* < 0.05 *vs*. vehicle-treated *ob/ob* mice; ^#^*P* < 0.05 *vs*. pair-fed wild type mice; ^$^*P* < 0.05 *vs*. pair-fed *ob/ob* mice.
