# Supplementary material for: Leptin administration restores the altered adipose and hepatic expression of aquaglyceroporins improving the non-alcoholic fatty liver of ob/ob mice
Source: Sci Rep. 2015 Jul 10;5:12067. doi: 10.1038/srep12067 (PMC4498231; doi:10.1038/srep12067)
Supplement: Supplementary Information [file srep12067-s1.pdf]

Leptin administration restores the altered adipose and hepatic expression of aquaglyceroporins improving the non-alcoholic fatty liver of *ob/ob* mice

Amaia Rodríguez[1,7,8], Natalia R. Moreno[3], Inmaculada Balaguer[1], Leire Méndez-Giménez[1,7,8], Sara Becerril[1,7,8], Victoria Catalán[1,7,8], Javier Gómez-Ambrosi[1,7,8], Piero Portincasa[4], Giuseppe Calamita[5], Graça Soveral[6], María M. Malagón[3,7], Gema Frühbeck[1,2,7,8,*]

[1]Metabolic Research Laboratory and [2]Department of Endocrinology & Nutrition, Clínica Universidad de Navarra, Pamplona, Spain; [3]Department of Cell Biology, Physiology, and Immunology, Instituto Maimónides de Investigación Biomédica (IMIBIC)/Reina Sofia University Hospital/University of Córdoba, Córdoba, Spain; [4]Clinica Medica "A. Murri", Department of Biomedical Sciences and Human Oncology University of Bari Medical School, Policlinico Hospital, Bari, Italy; [5]Department of Biosciences, Biotechnologies and Biopharmaceutics, University of Bari "Aldo Moro", Bari, Italy; [6]Research Institute for Medicines (iMed.ULisboa), Faculty of Pharmacy, Universidade de Lisboa, Lisboa, Portugal; [7]CIBER Fisiopatología de la Obesidad y Nutrición, Instituto de Salud Carlos III, 28029, Madrid, Spain; [8]Obesity & Adipobiology Group, Instituto de Investigación Sanitario de Navarra (IdiSNA), Pamplona, Spain.

***Corresponding author**:

Gema Frühbeck RNutr, MD, PhD
Dept. Endocrinology & Nutrition
Clínica Universidad de Navarra
Avda. Pío XII 36
31008 Pamplona (SPAIN)
Phone: +34 948 25 54 00 (ext. 4484)

Supplementary table S1. Sequences of primers and TaqMan® probes.

Gene (GenBank accesion no.)	Oligonucleotide sequence (5’-3’)	Nucleotides
Aqp3 (NM_016689.2)		
Forward	CCCTCTGGACACTTGGACATG	525-545
Reverse	TACTGGCCATCGTTGACCCT	598-617
Probe	FAM-CTTTGATCAGTTCATAGGCACA-TAMRA	557-578
Aqp7 (NM_007473.4)		
Forward	TCAGTTCCTGGGCTCCTTCTC	749-769
Reverse	CCTTGGAACCTGTCACCAACA	832-844
Probe	FAM-CCACCTACTTAATTTTCTATGGTGCC-TAMRA	781-806
Aqp9 (NM_022026.2)		
Forward	CTATGACGGACTCATGGCCTTT	578-599
Reverse	ATGAACGCCGTTCCATTTTCT	626-646
Probe	FAM-CTGATGGAAAGCTGCTC-TAMRA	601-617
Pparg (NM_001127330.1)		
Forward	GCTTCCACTATGGAGTTCATGCTT	576-599
Reverse	ATCCGGCAGTTAAGATCACACCTA	655-678
Probe	FAM-AGGATGCAAGGGTTTTTTCCGA-TAMRA	604-625

Aqp, aquaporin; *Pparg*, peroxisome proliferator-activator receptor γ.
